# Anti-Osteoporotic Effect of *Lactobacillus brevis* AR281 in an Ovariectomized Mouse Model Mediated by Inhibition of Osteoclast Differentiation

**DOI:** 10.3390/biology11030359

**Published:** 2022-02-24

**Authors:** Jing Yu, Yiling Hang, Wenni Sun, Guangqiang Wang, Zhiqiang Xiong, Lianzhong Ai, Yongjun Xia

**Affiliations:** School of Health Science and Engineering, University of Shanghai for Science and Technology, Shanghai 200093, China; joyceyu26@126.com (J.Y.); 18851572933@163.com (Y.H.); 15720509860@163.com (W.S.); 1015wanggq@163.com (G.W.); xiongzq@hotmail.com (Z.X.); ailianzhong1@126.com (L.A.)

**Keywords:** gut microbiota, osteoporosis, *Lactobacillus brevis*, osteoclastogenesis, osteoclast differentiation

## Abstract

**Simple Summary:**

Osteoporosis-related fractures are among the most common complications found in postmenopausal adults, which results in considerable economic impacts. Most treatments for osteoporosis increase bone formation or decrease bone resorption. While estrogen replacement therapy may be the gold standard for the treatment of osteoporosis, it also carries an increased risk of cardiac events and strokes in women. Thus, it is important to seek a safe and effective treatment method. Gut homeostasis is demonstrably linked to bone health. Probiotics are widely known to modulate gut microbiota, but with large strain differences. Our findings showed a strain of *Lactobacillus brevis* AR281 with an anti-osteoporotic property in ovariectomized mice, which may provide a new way to prevent osteoporosis.

**Abstract:**

Osteoporosis is a global disease characterized by weakened bone microarchitecture, leading to osteoporotic fractures. Estrogen replacement therapy is the traditional treatment for osteoporosis but carries with it an increased risk of cardiac events. In search of a safe and effective treatment, we used *Lactobacillus brevis* AR281, which has anti-inflammatory properties, to conduct a 7-week experiment, investigating its inhibitory effects on osteoporosis in an ovariectomized (ovx) mouse model. The results demonstrated that AR281 significantly improved bone microarchitecture and biomechanical strength in ovx mice by attenuating bone resorption. AR281 significantly decreased the critical osteoclast activator, the ratio of the receptor activator for nuclear factor kappa B (NF-κB) ligand (RANKL) to osteoprotegerin, and pro-inflammatory osteoclastogenic mediators, such as IL-1, IL-6, and IL-17, which can increase the RANKL expression. Moreover, AR281 modulated intestinal microbiota in ovx mice increased the abundance of *Akkermansia*, which is responsible for the improvement of gut epithelial barrier integrity. In an in vitro trial, AR281 suppressed the number of osteoclasts differentiated from the osteoclast precursor RAW264.7 cells caused by RANKL through the tumor necrosis factor (TNF) receptor-associated factor 6 (TRAF6)/NF-κB/nuclear factor of activated T cells c1 (NFATc1) pathway. Therefore, AR281 may be a natural alternative for combating osteoporosis.

## 1. Introduction

Osteoporosis is a mineral and bone disorder characterized by the structural deterioration of the bone tissue and weakened bone quality, leading to a fragile skeleton and a high risk of osteoporotic fractures [1]. It affects approximately 200 million individuals worldwide. Women are more susceptible to osteoporosis than men due to menopause-induced estrogen deficiency. Women experience a period of accelerated bone loss during the menopausal transition and postmenopausal years, thereby increasing a woman’s risk of developing osteoporosis [2]. The process of bone remodeling includes osteoblastic bone formation and osteoclastic bone resorption [3]. Most available treatments for osteoporosis modulate the process of bone remodeling [1]. Hormone replacement therapy (HRT), as a gold standard for the treatment for postmenopausal women, is used to alleviate menopausal vasomotor symptoms and bone loss and fractures [4]. However, HRT may cause an increase in cardiac events and stroke rates, especially in those who started HRT when over 70 years of age [5]. Previous studies also reported a non-negligible risk of breast cancer and endometrial cancer after women used HRT [6,7]. Consequently, patients need additional drugs to alleviate the side effects of HRT, such as progestins, which can inhibit endometrial cancer [6]. Alternative therapies, including bisphosphonates and raloxifene, a selective estrogen receptor modulator, are used after the patient has developed symptoms of osteoporosis or osteoporotic fractures. While the former alternative therapy has been associated with joint and muscle pain and unusual fractures [8,9], the latter is considerably less potent than HRT, and its ability to prevent nonvertebral fractures still needs further research [10]. Therefore, there is a need for safe and effective treatments for osteoporosis, preferably with good prevention before accelerated bone loss occurs.

Several studies demonstrate that osteoporosis can result from intestinal inflammation, as observed in intestinal bowel disease (IBD) [11,12,13]. Patients with IBD exhibit low bone mass in the early stages of the disease, suggesting a relationship between bone loss and IBD [14]. Additionally, a few autoimmune and inflammatory diseases with large amounts of high-sensitivity C-reactive protein levels may also lead to bone loss [15,16]. Studies have reported that many inflammatory cytokines, including interleukin (IL)-1, IL-6, and tumor necrosis factor (TNF)-α, increase during aging and play a vital role in osteoporosis [17,18,19]. IL-1 has been regarded as a biomarker for predicting periodontal inflammation, which can lead to irreversible alveolar bone loss; its sensitivity and specificity are over 80% and 60%, respectively [20]. IL-17 as a potent mediator in inflammatory responses is essential for osteoclastogenesis and bone resorption in rheumatoid arthritis (RA) [21]. Moreover, osteoclast activators and inhibitors, receptor activators for nuclear factor kappa B ligand (RANKL), and osteoprotegerin (OPG) are also involved in chronic inflammatory conditions, such as inflamm-aging and RA [22]. These cytokines induce the differentiation of osteoclast precursors (OCPs) into osteoclasts, which in turn may contribute to the maintenance of inflammatory levels through their ability to promote immune inflammatory responses and recruit more immune cells, resulting in chronic inflammation. Furthermore, while estrogen is known to be critical to bone remodeling, a recent study has identified that it has additional regulatory effects regarding inflammatory responses [23]. Suppression of inflammation in the host can help alleviate estrogen deficiency-induced osteoporosis [24]. Therefore, postmenopausal osteoporosis should be considered a result of an inflammatory disease, rather than merely a metabolic and endocrinologic condition.

Intestinal flora plays a vital role in human health and participates in the control of the host’s immune system. Numerous studies have demonstrated that the gut microbiota can modulate inflammatory diseases, including allergic inflammation, obesity, and RA [25,26,27,28]. Probiotics are live microbial feed supplements that, when administered in adequate amounts, regulate the host’s intestinal inflammation. In this study, we hypothesized that one such strain, *Lactobacillus brevis* AR281, would have an anti-osteoporotic effect due to its anti-inflammatory properties, which were reported in our pre-experiment [29]. *L. brevis* is a gram-positive bacterium found in fermented food and beverages, such as cheese, sauerkraut, kefir, and beer; it may also be found in the intestinal tracts of humans and animals [30,31]. *L. brevis* has been widely supplemented in yogurts and its safety has been widely confirmed [31,32]. The aims of our research were to assess the anti-osteoporotic effects of *L. brevis* AR281 in ovariectomized (ovx) mice and to explore its mechanism both in vivo and in vitro.

## 2. Materials and Methods

### 2.1. Animal Study

A total of 23 female C57BL/6 mice (8 weeks, specific-pathogen-free) were obtained from the JSJ Laboratory Animal Company (Shanghai, China) and were reared in the Laboratory Animal Center of Jiangnan University (Wuxi, China). The feeding conditions and surgical protocols were consistent with those described in our previous study [33]. Briefly, sixteen mice were subjected to the ovx operation, whereas seven mice were treated with a sham operation as controls. After a 1-week post-surgery recovery, all mice were of normal body weight and mental status. Their wounds showed no signs of infection and were gradually healing. After which, we divided the mice which underwent the ovx operation into the following two groups using random number generators (SPSS software version 23): OVX group (*n* = 8); *L. brevis* AR281-treated mice (AR281 group, *n* = 8). Mice that underwent the sham operation were labeled as SHAM group (*n* = 7). Mice in the AR281 group were treated with an oral gavage of AR281 (10^9^ CFU/mL) suspended in normal saline, whereas mice in other groups were treated with equivoluminal sterile normal saline on a daily basis for 7 weeks.

### 2.2. Detection of Biochemical Parameters, Fluorescein Isothiocyanate-Dextran and Endotoxin

Levels of bone alkaline phosphatase (BALP) and endotoxin in serum were detected by commercial kits (TW Reagent). The detection and calculation method of creatinine and deoxypyridinoline (DPD) and fluorescein isothiocyanate-dextran (FITC) were consistent with those of our previous study [33].

### 2.3. Micro-Computed Tomography and Biomechanical Detection

The left distal femoral bone mineral density (BMD), trabecular bone volume to tissue volume ratio (BV/TV), trabecular thickness (Tb.Th), trabecular number (Tb.N), and trabecular space (Tb.Sp) were calculated after three-dimensional morphometric quantification. The biomechanical properties (bending stress, bending load, and Young’s bending modulus) of femoral diaphysis were measured by a texture analyzer [34]. The test conditions of the micro-CT and biomechanics have been described in our previous study [33].

### 2.4. Cell Culture and Induction

The culture conditions of RAW264.7 macrophage cells were consistent with the previous study [35]. RAW264.7 cells were seeded in 12-well plates (10^4^ cells/well) and added with 50 ng/mL RANKL for inducing differentiation. The differentiated cells were co-cultured with different concentrations of AR281 on day 3. The study was divided into the following four groups: cell culture without RANKL; cell culture with 50 ng/mL RANKL; cell culture with 50 ng/mL RANKL and 10^5^ CFU/well AR281; cell culture with 50 ng/mL RANKL and 10^6^ CFU/well AR281. The cell medium was replaced by a fresh one, containing the same concentration of AR281, on day 4. Cells were fixed and stained on day 5.

### 2.5. TRAP Staining

Tartrate-resistant acid phosphatase (TRAP) staining was used to assess the osteoclast differentiation and the percentage of TRAP-positive multinucleated cells was calculated. Cells were stained for TRAP using a TRAP kit (Jiancheng, Nanjing, China). TRAP-positive multinucleated cells (>3 nuclei) were counted in eight fields of view for each sample and regarded as osteoclasts. Cells were observed using a Leica DM4 B microscope at ×200 magnification and images were captured and analyzed using equipment supplied by Leica Biosystems.

### 2.6. Cell Counting Kit-8 Assay

Raw264.7 cells were seeded in 96-well plates (10^4^ cells/well). Ten microliters of Cell Counting Kit-8 (CCK-8) reagent (Beyotime Biotechnology, Shanghai, China) were added to each well. After incubation for 2 h, the results were detected at the 450 nm wavelength using a microplate reader (SpecpraMax X3).

### 2.7. RNA Extraction from Small Intestine, Tibia, Fecal Samples and RAW264.7 Cells

A 20 mm piece of the duodenum from each mouse was collected and submerged in 1 mL RNAiso (Takara, Beijing, China). The samples were stored in liquid nitrogen for 5 min. The right side of the complete tibia was collected from each mouse, without the adhering flesh or tendons, and immediately stored at −80 °C. Fecal samples (>0.8 g per mouse) were collected before spoiling and immediately stored at −80 °C. RAW264.7. Cells were digested with trypsin (WISENT, Nanjing, China), and the medium was removed. RNA isolation and cDNA synthesis have been described in our previous study [33].

### 2.8. Real-Time Quantitative Polymerase Chain Reaction, Mus Primers, and High-Throughput 16SrDNA Sequencing

The primers used were those with proven validation on Primer Bank (Table S1). The relative gene expression was calculated by 2^−Δ^^CT^ method. The detailed information of cDNA amplification and 16SrDNA sequencing were consistent with those of our previous study [33].

### 2.9. Statistics

Data, including skeletal parameters, serum and urinary biochemical parameters, and the expression of cytokines in vivo and in vitro were shown as mean ± SEM. Preparation of figures and illustrations and the details of our data analyzing method have been reported in our previous study [33].

## 3. Results

### 3.1. Effects of L. brevis AR281 on Skeletal Parameters and Biomechanics

**Figure 1:**
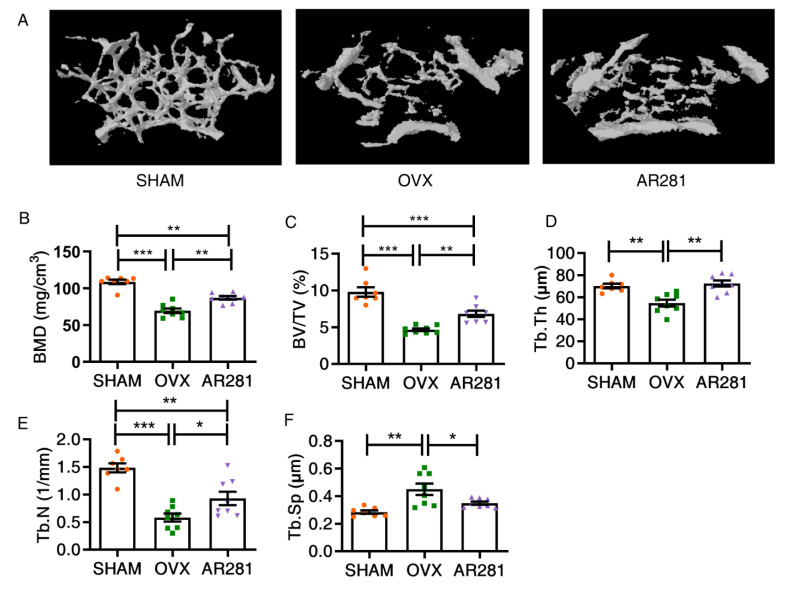
The effects of *L. brevis* AR281 on bone quality: (**A**) three-dimensional images of distal femur; (**B**) BMD; (**C**) BV/TV; (**D**) Tb.Th; (**E**) Tb.N; (**F**) Tb.Sp. Data are shown as mean ± SEM (*n* = 8 per group, except *n* = 7 in the SHAM group) and are normally distributed. * *p* < 0.05, ** *p* < 0.01, and *** *p* < 0.001.

To assess the anti-osteoporotic effects of AR281 on ovx mice, femurs were scanned to analyze BMD and bone microarchitecture using a micro-CT. Data were normally distributed according to the Shapiro–Wilk normality test. There was a significant increase (*p* < 0.05) in BMD, BV/TV, Tb.Th, and Tb.N and a significant reduction in the Tb.Sp of ovx mice, compared with those of mice not treated with ovx (Figure 1). However, oral gavage of AR281 in ovx mice showed a significant increase (*p* < 0.01) in BV/TV, Tb.Th, and BMD by 46.6%, 32.7%, and 25.3%, respectively, compared with those of ovx mice not treated with probiotics. AR281 treatment exhibited a significant increase (59.8%, *p* < 0.05) in Tb.N, and a significant reduction (22.4%, *p* < 0.05) in Tb.Sp compared with those of ovx mice not treated with probiotics. A biomechanical assay of the femur samples was performed to assess the effects of AR281 on the prevention of fractures. There was a significant enhancement (*p* < 0.05) in Young’s bending modulus of ovx mice, compared with that of mice not treated with ovx (Table 1). However, oral gavage of AR281 in ovx mice significantly increased the bending strength (*p* < 0.05) in Young’s bending modulus (*p* < 0.01) compared with those of ovx mice not treated with probiotics. Therefore, supplementation of AR281 increased bone mass and bone-breaking strength in ovx mice.

### 3.2. Effects of L. brevis AR281 on the Integrity of Gut Epithelial Barrier

**Figure 2:**
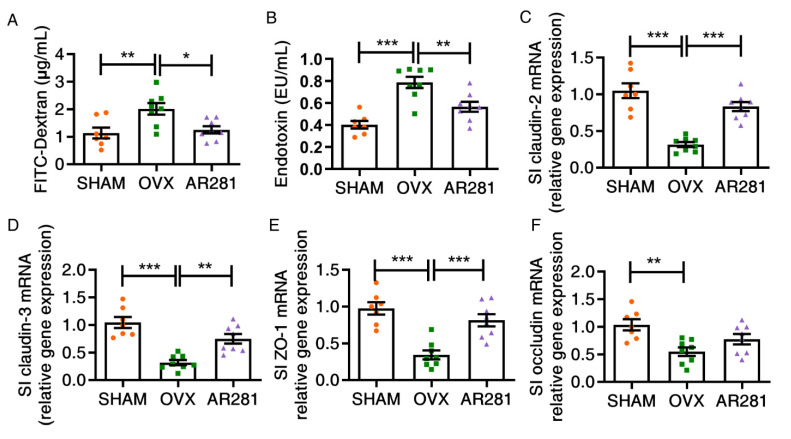
The effects of *L. brevis* AR281 on the integrity of gut epithelial barrier: (**A**) the levels of FITC-dextran in serum; (**B**) the contents of Endotoxin in serum; (**C**–**F**) the expression of claudin-2 and claudin-3, ZO-1, and occludin. Data are shown as mean ± SEM (*n* = 8 per group, except *n* = 7 in the SHAM group) and are normally distributed. * *p* < 0.05, ** *p* < 0.01, and *** *p* < 0.001.

The effect of *L. brevis* AR281 on the gut epithelial permeability of ovx mice was investigated via the detection of the fluorescein isothiocyanate (FITC) dextran and serum endotoxin (Figure 2A,B). There was a significant increase in the levels of serum FITC-dextran (*p* < 0.01) and endotoxin (*p* < 0.001) of ovx mice, compared with those of mice not treated with ovx. However, oral gavage of AR281 in ovx mice significantly suppressed the levels of FITC-dextran (*p* < 0.05) and endotoxin (*p* < 0.01), compared with those of ovx mice not treated with probiotics. Moreover, ovx mice exhibited significantly downregulated (*p* < 0.01) expression levels of tight junction proteins, including claudin-2, claudin-3, ZO-1, and occludin (Figure 2C–F). However, oral gavage of AR281 in ovx mice exhibited significantly upregulated expression profiles of the first three factors (*p* < 0.01), compared with those of ovx mice not treated with probiotics. Therefore, AR281 exhibited a protective effect on the intestinal barrier integrity in ovx mice.

### 3.3. Effects of L. brevis AR281 on Intestinal Inflammatory Responses and Intestinal Flora

**Figure 3:**
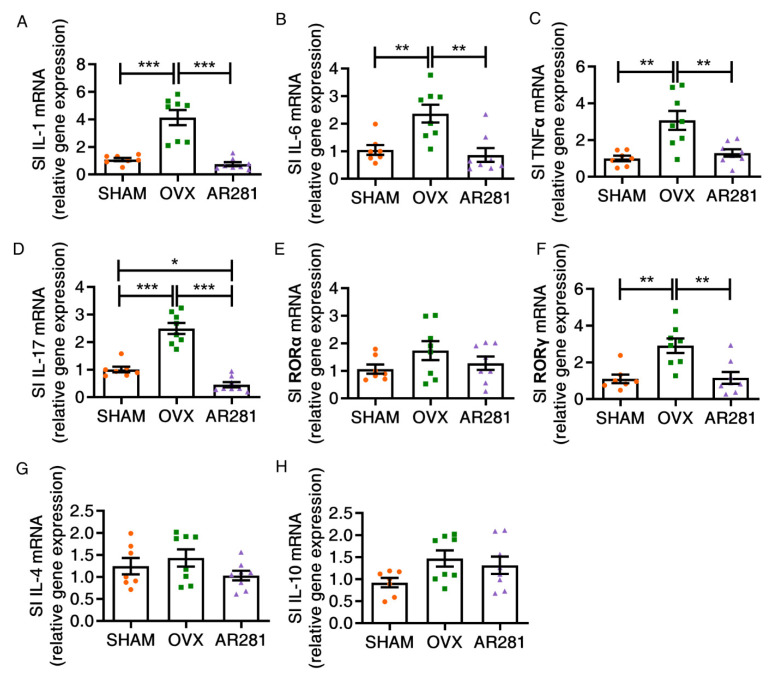
The effects of *L. brevis* AR281 on intestinal inflammation: (**A**–**D**) The expression of IL-1, IL-6, TNFα, and IL-17; (**E**,**F**) the expression of IL-17-related transcript factors RORα and RORγ; (**G**,**H**) the expression of anti-inflammatory cytokines IL-4 and IL-10. Data are shown as mean ± SEM (*n* = 8 per group, except *n* = 7 in the SHAM group) and are normally distributed. * *p* < 0.05, ** *p* < 0.01, and *** *p* < 0.001.

To assess the levels of intestinal inflammation, we examined the expression profiles of intestinal proinflammatory and anti-inflammatory cytokines. There was a significant increase (*p* < 0.01) in small intestinal proinflammatory cytokines in ovx mice, including IL-1, IL-6, and TNFα, compared with those of mice without ovx (Figure 3A–C). However, oral gavage of AR281 in ovx mice significantly suppressed the levels of IL-1, IL-6, and TNFα (*p* < 0.01) compared with those of ovx mice not treated with probiotics. There was a significant increase in small intestinal IL-17-related proinflammatory cytokines in ovx mice, including IL-17 and RORγ, compared with those of mice not treated with ovx, but the level of RORα did not show a significant change between the two groups (Figure 3D–F). AR281 treatment significantly inhibited the expression of IL-17 (*p* < 0.001) and RORγ (*p* < 0.01) in the small intestine compared with those of ovx mice not treated with probiotics. However, oral gavage of AR281 in ovx mice exhibited lower expression profiles of IL-17 (*p* < 0.05) in the small intestine than mice not treated with ovx. Supplementation of AR281 did not change the expression of RORα in ovx mice. Additionally, there was no significant change in anti-inflammatory cytokines in the small intestine after different treatments, including IL-4 and IL-10 (Figure 3G,H). Overall, AR281 treatment significantly suppressed inflammatory responses in the small intestine.

**Figure 4:**
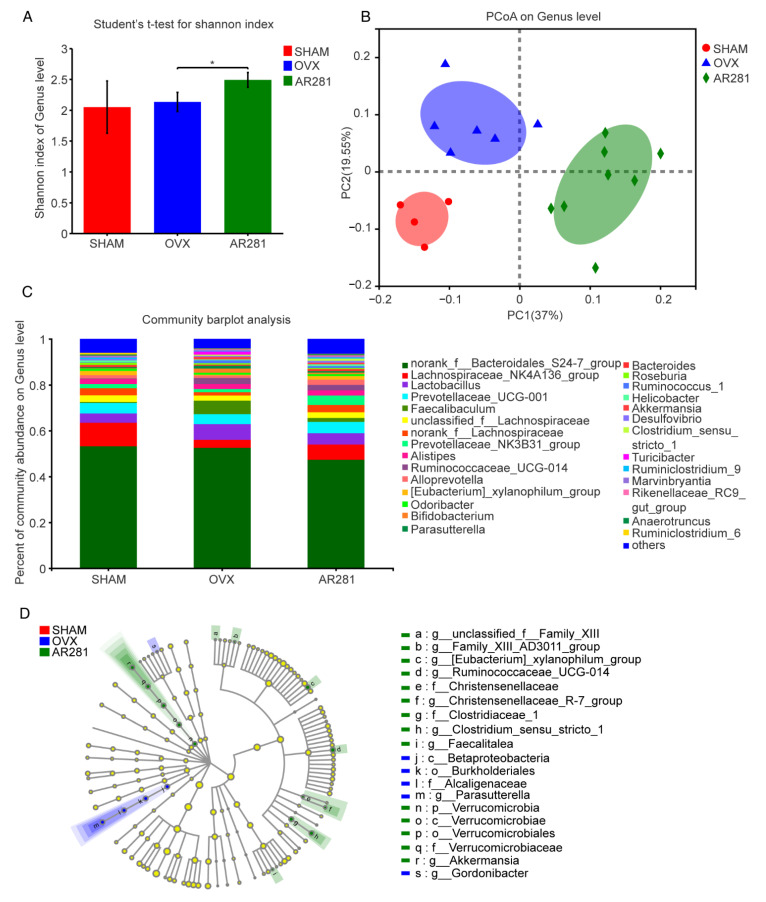
The effects of *L. brevis* AR281 on intestinal microbial variation: (**A**) Shannon index of the genus level; (**B**) principal coordinates analysis (PCoA) plots of fecal microbial communities based on abundance Jaccard distance; (**C**) community bar-plot analysis of the fecal microbiota; (**D**) linear discriminant analysis effect size (LEfSe) of fecal microbial communities among groups. *n* = 4, 6, or 8 in SHAM, OVX, or AR281, respectively. * *p* < 0.05.

To further investigate the mechanism by which AR281 inhibited pro-inflammatory cytokines, we examined the fecal microbial variation in mice. At the level of genus, the α-diversity of the fecal microbial communities of ovx mice treated with AR281 was significantly higher (*p* < 0.05) than that of ovx mice not treated with probiotics (Figure 4A). The principal coordinate analysis (PCoA) indicated that the structure of fecal microbial communities in three experimental groups was significantly changed (Figure 4B,C). Linear discriminant analysis effect size (LEfSe) showed that the abundances of the genus *Akkermansia* and the family Verrucomicrobiaceae, the order Verrucomicrobiales, the class Verrucomicrobiae, and the phylum Verrucomicrobia, which *Akkermansia* belongs to, were significantly higher in the AR281 group, whereas the abundances of the genus *Parasutterella* and the family Alcaligenaceae, the order Burkholderiales, and the class Betaproteobacteria, which *Parasutterella* belongs to, were significantly higher in the ovx mice (Figure 4D).

### 3.4. Effects of L. brevis AR281 on Bone Metabolism

**Figure 5:**
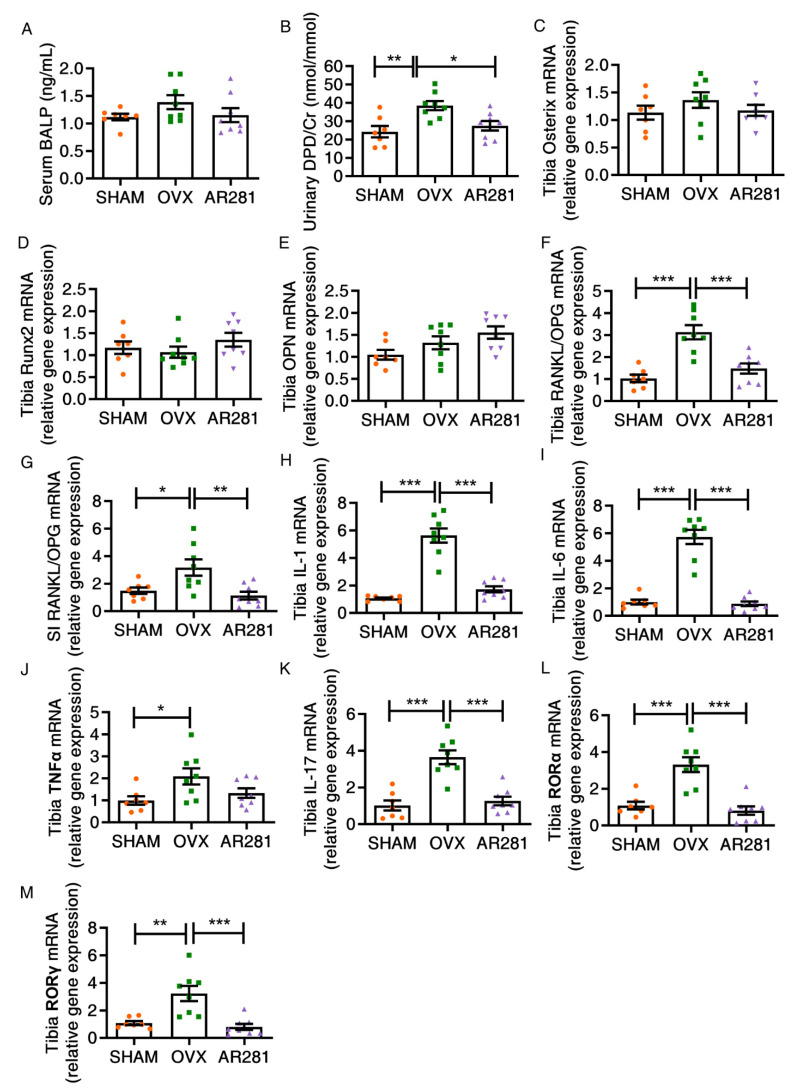
The effects of *L. brevis* AR281 on osteoclastogenic signaling in the tibia: (**A**) bone alkaline phosphatase (BALP) in serum; (**B**) the ratio of urinary deoxypyridinoline (DPD) to creatinine; (**C**–**E**) the bone formation-related transcript factors or protein osterix, Runx2, and osteopontin (OPN) in the tibia; (**F**,**G**) the RANKL/OPG ratio in the bone marrow of the tibia and small intestine; (**H**–**K**) the expression of IL-1, IL-6, TNFα, and IL-17; (**L**,**M**) the expression of RORα and RORγ. Data are shown as mean ± SEM (*n* = 8 per group, except *n* = 7 in the SHAM group) and are normally distributed. * *p* < 0.05, ** *p* < 0.01, and *** *p* < 0.001.

We examined the biomarkers of bone formation and resorption (Figure 5A,B). The marker of bone formation BALP did not change after different treatments. However, ovx mice exhibited a significant increase in the bone resorption marker DPD compared with that of mice not treated with ovx. AR281 treatment significantly inhibited (*p* < 0.05) ovx-induced increase in DPD. Moreover, the treatments failed to affect the expression of factors related to bone formation, including osterix, Runx2, and osteopontin (OPN), among the different groups (Figure 5C–E). However, there was a significant growth in the osteoclastogenic activator ratio of RANKL to OPG (RANKL/OPG), both in the bone marrow (*p* < 0.05) and small intestine (*p* < 0.001) of ovx mice, compared with those of mice not treated with ovx (Figure 5F,G). AR281 treatment significantly inhibited RANKL/OPG in the tibia (*p* < 0.001) and the small intestine (*p* < 0.01) compared with that of ovx mice not treated with probiotics. Furthermore, ovx mice exhibited significantly increased levels (*p* < 0.05) of osteoclastogenic cytokines in the tibia, including IL-1, IL-6, and TNFα, compared with those of mice not treated with ovx (Figure 5H–J). However, oral gavage of AR281 in ovx mice exhibited a decrease (*p* < 0.001) in the production of IL-1 and IL-6 in the tibia, compared with those of ovx mice not treated with probiotics. Oral gavage of AR281 in ovx mice failed to significantly affect the expression of TNFα in the tibia. Moreover, ovx mice exhibited significantly increased levels (*p* < 0.01) of IL-17-related pro-osteoclastogenic cytokines in the tibia, including IL-17, RORα, and RORγ, compared with those of mice not treated with ovx (Figure 5K–M). However, oral gavage of AR281 in ovx mice significantly inhibited (*p* < 0.001) the three factors of the tibia, compared with those of ovx mice not treated with probiotics. Overall, supplementation of AR281 significantly suppressed osteoclastogenic cytokines in both the small intestine and tibia.

### 3.5. Effects of AR281 on RANKL-Induced Osteoclastogenesis in RAW264.7 Cells

**Figure 6:**
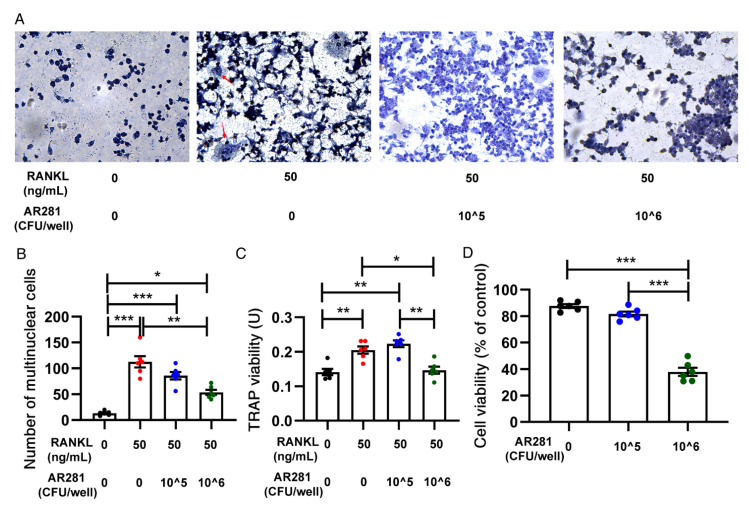
The effect of *L. brevis* AR281 on osteoclast differentiation from RAW264.7 cells: (**A**,**B**) the number of multinucleated cells under a light microscope (original magnification ×200); (**C**) the tartrate-resistant acid phosphatase (TRAP) activity; (**D**) the viability of RAW264.7 cells when they were co-cultured with different concentrations of AR281. Data are shown as mean ± SEM (*n* = 6) and are normally distributed. * *p* < 0.05, ** *p* < 0.01, and *** *p* < 0.001.

**Figure 7:**
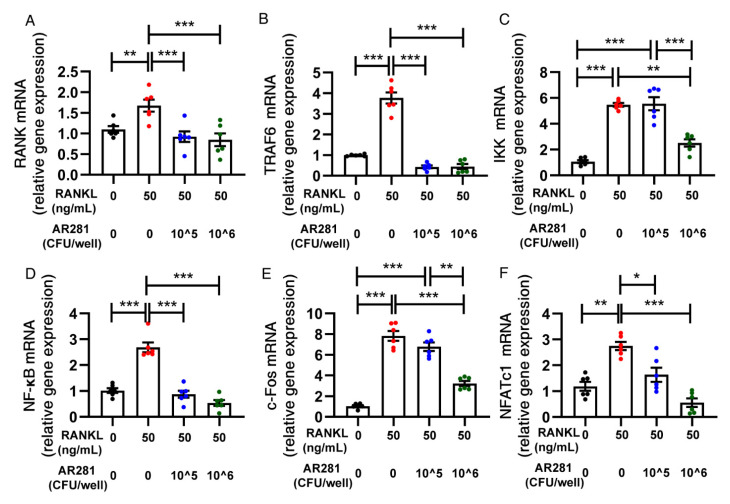
The effects of *L. brevis* AR281 on the expression profiles of osteoclast differentiation-related markers: (**A**) the expression of RANK; (**B**) TRAF6; (**C**) IKK; (**D**) NF-κB; (**E**) c-Fos; (**F**) NFATc1. Data are shown as mean ± SEM (*n* = 6) and are normally distributed. * *p* < 0.05, ** *p* < 0.01, and *** *p* < 0.001.

To further investigate the mechanism by which AR281 inhibited osteoclastogenesis, we used RANKL to stimulate the differentiation of RAW264.7 cells into osteoclasts. We first examined its effects on the formation of osteoclast-like multinucleated cells (>3 nuclei) and TRAP activity in RAW264.7 cells (Figure 6A–C). TRAP staining showed that there were more multinucleated cells when RAW264.7 cells were stimulated by RANKL. However, AR281 significantly attenuated the formation of osteoclast-like multinucleated cells (*p* < 0.01) at a concentration of 10^6^ CFU/well, rather than 10^5 CFU/well. Moreover, AR281 (10^6^ CFU/well) significantly inhibited RANKL-induced TRAP activity (*p* < 0.01); however, TRAP activity significantly decreased when the concentration of AR281 was 10^6^, rather than 10^5^ CFU/well (*p* < 0.01). These inhibitory effects on osteoclast differentiation were not due to the cytotoxicity of AR281, as the cell viability of RAW264.7 cells did not significantly change when co-cultured with the 10^5^ and 10^6^ CFU/well of AR281 (Figure 6D). However, cell viability was significantly reduced when the concentration was 10^7^ CFU/well; therefore, we did not consider the results of this concentration in our study. Additionally, we investigated the molecular mechanism by which AR281 inhibited osteoclastogenesis. AR281 significantly inhibited (*p* < 0.001) RANKL-induced expression of RANK and tumor necrosis factor receptor-associated factor 6 (TRAF6) at concentrations of 10^5^ and 10^6^ CFU/well (Figure 7A,B). AR281 significantly inhibited (*p* < 0.01) the RANKL-induced expression of inhibitor of nuclear factor kappa-B (NF-κB) kinase (IKK) at a concentration of 10^6^ CFU/well; however, the expression level was significantly lower (*p* < 0.001) when the concentration of AR281 was 10^6^, rather than 10^5^ CFU/well (Figure 7C). Moreover, AR281 significantly inhibited (*p* < 0.001) RANKL-induced expression of NF-κB at concentrations of 10^5^ and 10^6^ CFU/well (Figure 7D). Additionally, AR281 significantly inhibited (*p* < 0.001) RANKL-induced expression of c-Fos at a concentration of 10^6 CFU/well; however, the expression level was significantly lower (*p* < 0.01) at a concentration of 10^6^, rather than 10^5^ CFU/well of AR281 (Figure 7E). Moreover, AR281 significantly suppressed the expression of nuclear factor of activated T cells c1 (NFATc1) at concentrations of 10^6^ CFU/well (*p* < 0.001) and 10^5 CFU/well (*p* < 0.05); however, the expression level was not significantly lower when the concentration of AR281 was 10^6^, rather than 10^5^ CFU/well (Figure 7F). Overall, AR281 exhibited better inhibition of osteoclastogenesis at a concentration of 10^6^ than at 10^5^ CFU/well.

## 4. Discussion

We confirmed the anti-osteoporotic properties of *L. brevis* AR281, as shown by improved cancellous bone tissue and enhanced bone-breaking strength. This strain alleviated osteoporosis by significantly reducing bone resorption. Moreover, we explored the inhibitory effects of AR281 on bone resorption in the following two stages: (1) the effects of AR281 on the RANKL/OPG ratio and (2) the effects of AR281 on the differentiation of OCPs into osteoclasts after the binding of RANKL and RANK. In an in vivo trial, we confirmed that AR281 suppresses the osteoclast activator RANKL/OPG and related pro-inflammatory osteoclastogenic cytokines, which can stimulate the production of RANKL. In an in vitro trial, AR281 suppressed the differentiation of OCPs into osteoclasts via the TRAF6//NF-κB/NFATc1 pathway. Several strains of *L. brevis* have anti-inflammatory properties [36,37,38], but studies on the effects of *L. brevis* on ovx-induced osteoporosis are still limited.

Gut microbiota has been demonstrated to play a critical role in osteoporosis caused by estrogen deficiency [39]. Bone loss did not occur when female mice lacked both gut microbiota and estrogen. The levels of biomarkers of bone remodeling in estrogen-deficient germ-free (GF) mice did not change compared with those in normal GF mice but increased when GF mice were colonized with the intestinal microbiota of normal mice [24], suggesting that intestinal microbiota is involved in the modulation of estrogen deficiency-induced osteoporosis. In our study, AR281 exhibited a great capacity for modulating the gut microbiota in ovx mice, as shown by the decreased amounts of *Parasutterella* and higher amounts of *Akkermansia* in the feces of mice. Studies have reported that the amount of *Parasutterella* (*Proteobacteria*) is much higher in intestinal mucosal tissue and feces in Crohn’s disease samples [40,41], and might be associated with increased intestinal inflammation in the host. *Akkermansia* is the only member of the *Verrucomicrobia* phylum and has been regarded as a novel microbe with probiotic properties [42]. Several studies have provided evidence that *Akkermansia* can improve mucus thickness in the inner layer, enhance the number of goblet cells, and promote the expression profiles of tight junction proteins of epithelial cells, such as the claudin-family, ZO-1, and occludin, in obese mice and alcohol-induced fatty liver mice [43,44]. The development of IBD is negatively associated with the abundance of *Akkermansia* [45]. Another study showed that high-fat diet-induced pro-inflammatory cytokines, including IL-1 and IL-6, are reversed by *Akkermansia* through Treg cells [46]. Therefore, we speculated that the increased abundance of *Akkermansia* might be responsible for improved integrity of intestinal epithelial barrier and reduced intestinal inflammatory responses and bone resorption in the mice supplemented with AR281. However, the type of intestinal microbial profile that can help reduce bone resorption and alleviate osteoporosis has not been determined, necessitating further research to elucidate upon this.

Bone resorption by osteoclasts is predominantly mediated by the RANKL/OPG/RANK system. The binding of RANK and its specific receptor RANKL is essential for the differentiation and development of osteoclasts. OPG, a competitor receptor of RANKL, plays a vital role in suppressing osteoclastogenesis by combining with RANKL, thereby suppressing the combination of RANKL and RANK [47]. Therefore, the inhibition of the RANKL/OPG ratio prevents bone resorption. Consistent with the results of our study, AR281 treatment attenuated this ratio and exhibited an anti-osteoclastogenic effect. Moreover, there is a complex network involving different proinflammatory cytokines in modulating this ratio, which has been reported in our previous study [33]. Briefly, TNFα and IL-1 can promote osteoclastogenesis indirectly by inducing the RANKL expression and act directly on OCPs to differentiate into osteoclasts. IL-6 increases osteoclastogenesis by inducing the production of IL-1 and RANKL. IL-17 promotes the expression of IL-1, IL-6, TNFα, and RANKL, but inhibits OPG expression in the osteoblast lineage. The absence of transcription factors RORα and RORγ can result in decreased expression of IL-17. Zhou and colleagues reported that *Lactobacillus rhamnosus* GG (LGG) prevented excessive bone resorption by inhibiting the expression of IL-17 and RANKL in bone marrow [48]. Kim and colleagues showed that *Lactobacillus*-fermented milk alleviated bone loss by reducing the production of the pro-inflammatory cytokines TNFα and IL-1 [49]. Similar to our results, AR281 exhibited an inhibitory effect on the production of IL-1, IL-6, and IL-17, which might be responsible for an inhibitory effect on the ratio RANKL/OPG. However, AR281 did not modulate the inhibitory cytokines of bone resorption, including IL-4 and IL-10. Therefore, AR281 inhibited the RANKL/OPG ratio mainly through the suppression of proinflammatory osteoclastogenic cytokines, rather than by modulating anti-inflammatory cytokines.

Consistent with these in vivo results, our in vitro experiments demonstrated a potential direct role of *L. brevis* AR281 in suppressing osteoclast differentiation in RANKL-induced RAW264.7 cells, which are widely known as OCPs and are considered as a key cell line for in vitro analyses [35]. As a result of RANKL binding, RANK activates the expression of TRAF6, which keys to the differentiation of OCPs into osteoclasts. TRAF6 decoy peptides attenuate the differentiation and development of osteoclasts [50]. Our study revealed that AR281 reversed the number of osteoclast-like multinucleated cells and TRAP activity induced by RANKL stimulation. RANKL increased the expression of TRAF6, whereas AR281 inhibited this effect. TRAF6 is a signal transducer in the NF-κB pathway, which can stimulate the expression of IKK to mediate inflammatory responses [51]. NF-κB regulates RANKL-induced OCP differentiation by activating c-Fos and NFATc-1 [52]. NFATc-1 is an osteoclast-specific transcription factor that modulates osteoclast-specific gene expression [53]. The expression of c-fos induces the expression of NFATc1, which is critical to osteoclast differentiation and development. Lim and colleagues confirmed that the extract of *Lactobacillus casei* attenuated the differentiation of RAW264.7 cells by modulating the NF-κB pathway, which downregulates the expression of c-fos and NFATc-1 [54]. Similar to our results, the RANKL-induced increase in the expression of IKK, NF-κB, c-fos, and NFATc1 was reversed by AR281. Overall, AR281 not only regulates the osteoclast activator RANKL/OPG by suppressing proinflammatory osteoclastogenic cytokines, but it also inhibits the differentiation of OPCs into osteoclasts through the TRAF6/NF-κB/NFATc-1 pathway after RANKL and RANK are combined.

## 5. Conclusions

In summary, *L. brevis* AR281 reconstructed the intestinal microbiota of ovx mice and exhibited an anti-osteoporotic effect by alleviating bone resorption, rather than regulating bone formation. AR281 suppressed the RANKL/OPG ratio by downregulating the expression of pro-inflammatory osteoclastogenic cytokines, thereby inhibiting the binding of RANKL and RANK on OCPs. AR281 further attenuated the osteoclast differentiation by modulating the TRAF6/NF-κB/NFATc1 pathway after the binding of RANKL and RANK.

## Figures and Tables

**Table 1:** Effects of *L. brevis* AR281 on bone-breaking strength.

	Bending Stress (MPa)	Bending Load (N)	Young’s Bending Modulus (MPa)
SHAM	357.84 ± 22.65	14.05 ± 0.89	29,834.95 ± 1888.83 *
OVX	338.75 ± 13.92	13.30 ± 0.55	25,263.29 ± 2572.43
AR281	378.38 ± 23.64 *	14.86 ± 0.93	31,743.22 ± 3073.48 **

Data are shown as mean ± SEM (*n* = 8 per group, except *n* = 7 in the SHAM group) and are normally distributed. * *p* < 0.05 and ** *p* < 0.01 vs. OVX group.

## Data Availability

RAW264.7 cell line was obtained from BeNa Culture Collection (No. BNCC337875). The sequence dataset associated with this paper is available in the NCBI SRA repository under BioProject ID: SUB10967810.

## Supplementary Materials

The following supporting information can be downloaded at https://www.mdpi.com/article/10.3390/biology11030359/s1, Table S1: Primers sequences for quantitative Real-Time polymerase chain reaction.
