# Supplementary material for: Anti-Osteoporotic Effect of Lactobacillus brevis AR281 in an Ovariectomized Mouse Model Mediated by Inhibition of Osteoclast Differentiation
Source: Biology (Basel). 2022 Feb 24;11(3):359. doi: 10.3390/biology11030359 (PMC8944959; doi:10.3390/biology11030359)
Supplement: Supplementary file 1 [file biology-11-00359-s001.zip › biology-1587696-supplementary.pdf]

Table S1. Primers sequences for quantitative Real-Time polymerase chain reaction.

Primers	Forward Primer (5′–3′)	Reverse Primer (5′–3′)
HPRT1	GAAGGAGATGGGAGGCAATCACATT	AATCCAGCAGGTCAGCAAAGAACTT
TNFα	AGGGTCTGGGCCATAGAACT	CCACCACGCTCTTCTGTCTAC
claudin-2	ACCGTGTTCTGCCAGGAATCTC	CCAGGATGCCACCAAGGATGAA
claudin-3	TCATCGGCAGCAGCATCATCAC	CCAGCAGCGAGTCGTACATCTTG
ZO-1	CTTCTCTTGCTGGCCCTAAAC	TGGCTTCACTTGAGGTTTCTG
occludin	TTGAAAGTCCACCTCCTTACAGA	CCGGATAAAAAGAGTACGCTGG
TRAF6	AAAGCGAGAGATTCTTTCCCTG	ACTGGGGACAATTCACTAGAGC
IKK	ACAGCCAGGAGATGGTACG	CAGGGTGACTGAGTCGAGAC
NFATc1	GACCCGGAGTTCGACTTCG	TGACACTAGGGGACACATAACTG
NF-κB	ACACTGGAAGCACGGATGAC	TGTCTGTGAGTTGCCGGTCT
RANK	TGAGCCTCCGAGCAGAACTGAC	TGCCTGTGTAGCCATCTGTTGAGT
RANKL	GATGGAAGGCTCATGGTTGGATGTG	GGCAGCATTGATGGTGAGGTGTG
OPG	GCAGAGACGCACCTAGCACTGA	CGCAGCACAGCCACTTGTTCAT
IL-1	TCGCAGCAGCACATCAACAAGA	CCACGGGAAAGACACAGGTAGC
IL-6	TAGTCCTTCCTACCCCAATTTCC	TTGGTCCTTAGCCACTCCTTC
IL-17	CCACCTCACACGAGGCACAAGT	TCAGCAGCAGCAACAGCATCAGA
IL-4	GGTCTCAACCCCCAGCTAGT	GCCGATGATCTCTCTCAAGTGAT
IL-10	GCTCTTACTGACTGGCATGAG	CGCAGCTCTAGGAGCATGTG
RORα	GTGGAGACAAATCGTCAGGAAT	TGGTCCGATCAATCAAACAGTTC
RORγ	GACCCACACCTCACAAATTGA	AGTAGGCCACATTACACTGCT
Osterix	ATGGCGTCCTCTCTGCTTG	TGAAAGGTCAGCGTATGGCTT
Runx2	ATGCTTCATTCGCCTCACAAA	GCACTCACTGACTCGGTTGG
OPN	AGCAAGAAACTCTTCCAAGCAA	GTGAGATTCGTCAGATTCATCCG
